# Outcomes and Challenges of Optimal Fixation Selection for Metaphyseal Fixation Techniques in Revision Knee Arthroplasty According to Bone Defects: A Systematic Review

**DOI:** 10.7759/cureus.89060

**Published:** 2025-07-30

**Authors:** Wael S Alhifzi

**Affiliations:** 1 Orthopedics, Aseer Central Hospital, Ministry of Health, Abha, SAU

**Keywords:** aori classification, a systematic review, bone defect, bone loss, metaphyseal bone defect, metaphyseal cones, metaphyseal sleeves, revision total knee arthroplasty, rtka, total knee arthroplasty (tka)

## Abstract

Revision total knee arthroplasty (rTKA) is often complicated by metaphyseal bone loss, requiring stable fixation techniques to restore function and alignment. Metaphyseal sleeves and cones have shown promising clinical outcomes, particularly in cases of severe bone defects. However, no gold standard therapy has been established. Therefore, this review aimed to evaluate and compare the clinical and radiological outcomes of metaphyseal sleeves and cones in rTKA, considering the severity of bone defects and related complications.

This systematic review followed the Preferred Reporting Items for Systematic Reviews and Meta-Analyses (PRISMA) guidelines. Thirteen studies published between 2004 and 2024 were included, evaluating clinical and radiological outcomes of metaphyseal sleeves and cones across varying Anderson Orthopaedic Research Institute (AORI) defect classifications.

Metaphyseal sleeves demonstrated a 100% survival rate over five years across multiple studies, while metaphyseal cones showed a survivorship rate of 91 91%. Both techniques demonstrated satisfactory functional recovery, although outcome measurement tools varied across studies, such as the Knee Society Score (KSS), which showed progressive improvement in most patients in both groups. Sleeves were associated with a higher incidence of intraoperative fractures (3.7%), whereas cones required more re-revision surgeries (5.33%), often due to issues with cement fixation.

Both metaphyseal sleeves and cones provided effective fixation in rTKA. Neither method proved universally superior; the choice should be tailored to the severity of the defect and individual patient factors to balance durability, risk, and clinical outcome.

## Introduction and background

Total knee arthroplasty (TKA), also known as total knee replacement, is regarded as one of the most successful, economical, and predictable procedures in modern orthopedics. It is primarily indicated for patients with symptomatic and end-stage degenerative osteoarthritis who experience persistent pain and functional limitations that impair daily activities and reduce quality of life [1,2].

However, despite its widespread use and clinical success, increasing life expectancy and rising surgical procedures have created a greater demand for revision TKA (rTKA), typically due to complications such as bone loss, aseptic loosening, instability, stiffness, deep or superficial wounds, or periprosthetic joint infection (PJI) or fracture [3,4].

rTKA poses significant challenges, particularly when metaphyseal bone defects compromise implant stability [5]. The Anderson Orthopaedic Research Institute (AORI) classification system is used to assess the severity of bone loss, with types 2B and 3 recognizing the most complex defects [6]. Fixation options, chosen based on defect size, location, and type, include bone grafts, composites, wedges, and cemented or cementless metal implants, such as metaphyseal sleeves and cones [7].

Metaphyseal sleeves and cones have emerged as favored techniques due to their high porosity, which promotes osseointegration, while mid- to long-term studies demonstrate reliable performance and survivorship. Compared to other techniques, such as structural allografts or wedges, sleeves and cones provide enhanced mechanical stability and biological fixation, and are increasingly utilized in revision scenarios [8,9]. This approach aligns with the zonal fixation principle, which recommends achieving stable implant anchorage in at least two of the three anatomical zones, including epiphysis, metaphysis, and diaphysis, to maximize component survival in rTKA [10].

However, there is a lack of definite guidelines for metaphyseal fixation in rTKA, with no gold standard therapy considered [11]. Nonetheless, multiple mid-term follow-up studies were tackling the clinical and radiological outcomes and complications of different metaphyseal fixation techniques.

This systematic review addresses the growing need for rTKA driven by rising life expectancy and implant failures, in which metaphyseal bone defects critically compromise stability. Although metaphyseal sleeves and cones offer promising osseointegration and mechanical support, no consensus guidelines exist to direct their use. Therefore, this systematic review compares clinical and radiographic outcomes and associated complications of these fixation methods across varying defect severities.

## Review

Methods

Search Strategy

This systematic review followed the Preferred Reporting Items for Systematic Reviews and Meta-Analyses (PRISMA) guidelines [12]. The search was conducted through several databases, including PubMed, Medline, Scopus, and Web of Science. The full Boolean search string was: (Arthroplast* OR Knee) AND (Revision OR "revision total knee arthroplasty" OR "rTKA" OR "revision TKA") AND (bone OR defect OR loss OR "bone defect" OR "bone loss" OR type 2 OR type 3) AND ("Anderson classification" OR "AORI classification" OR "Anderson Orthopaedic Research Institute" OR "AORI") AND (metaphyseal OR Fixation OR "metaphyseal Fixation") AND (sleeve* OR cone* OR augment*).

Eligibility Criteria

This review included original English-language articles published between 2004 and 2024 that evaluated metaphyseal fixation techniques in rTKA. Studies were included if they reported clinical, radiographic, or patient-reported outcomes with a mean follow-up of three years. This threshold was selected to ensure sufficient time for evaluating mid-term implant performance and patient functionality.

Eligible study designs comprised retrospective and prospective observational studies, clinical trials, case-control studies, and case series. Excluded were non-English studies, publications prior to 2004, reviews, case reports, and studies lacking relevant outcomes, populations, or interventions.

Data Extraction and Synthesis

**Figure 1:**
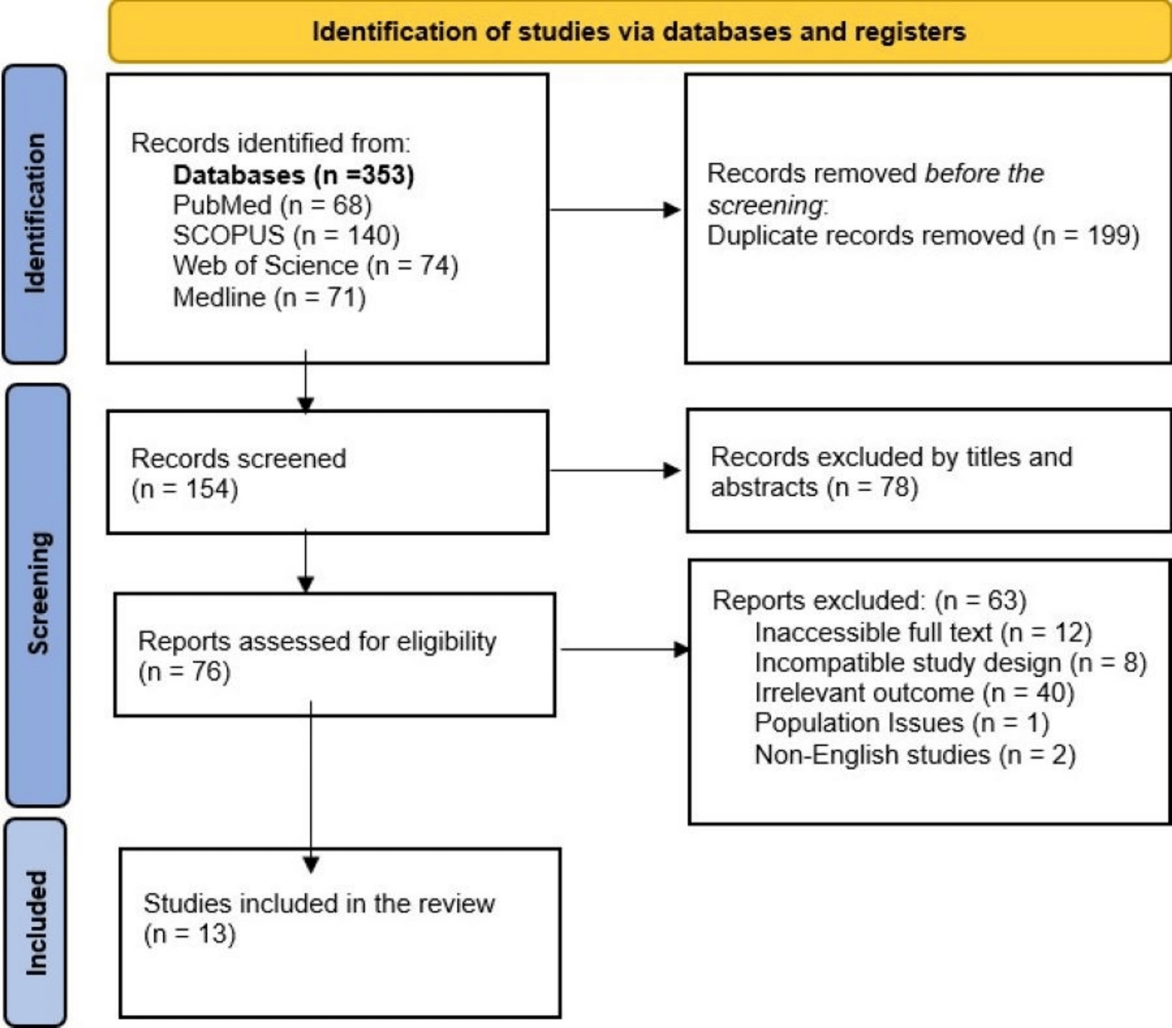
Flow diagram of study selection for the included studies in the systematic review.

Following PRISMA guidelines, two independent reviewers screened titles and abstracts and assessed full-text articles for eligibility. Discrepancies were resolved through discussion or adjudicated by a third author. Figure 1 outlines the study selection process, detailing how the initial pool was narrowed to 13 eligible studies based on predefined criteria.

After screening the article's title and abstract according to the eligibility criteria, two independent authors reviewed the full text. The following data were extracted from the potentially relevant articles: the first author’s last name, year of publication, country of origin, study aim, and study duration. Also, population characteristics included the number of patients, gender, mean age, mean body mass index (BMI), American Society of Anesthesiologists (ASA) grade, and AORI type. In addition to the intervention details, the mean follow-up years, revision knee location, fixation technique, functional assessment tool used, and indication for primary TKA and rTKA were noted. Outcomes included the % survival rate, functional outcome, and conclusion. Lastly, the intra-operative and postoperative complications related to the fixation technique used in the rTKA are also discussed, as well as the need for re-revision or re-operation.

Quality Assessment

We assessed the risk of bias for each included study using the appropriate tool based on the study design. Since all studies were retrospective and prospective observational, we used the Risk of Bias in Non-randomized Studies of Interventions (ROBINS-I) tool [13], which evaluates seven domains: confounding, selection bias, classification of intervention, deviation from the intended intervention, missing data, measurement of the outcomes, and selection of the reported results. The assessment was based on the overall risk as low, moderate, or serious, based primarily on how well studies addressed confounding and participant selection, two key sources of bias in observational designs. A visual summary of these assessments is provided below, generated using Robvis (Risk-of-Bias VISualization), which illustrates domain-level bias ratings and overall judgments across the included studies.

Statistical Analysis

All outcome data were summarized descriptively. Owing to substantial clinical and methodological heterogeneity across the included studies (variations in implant types, outcome definitions, and follow-up durations), no formal meta-analysis was performed; all data are presented descriptively. Future updates to this review should incorporate pooled effect estimates with 95% confidence intervals, forest plots, subgroup and sensitivity analyses, and adjustments for study-level risk of bias to strengthen inferential comparisons.

Results

Our systematic review comprised a total of 13 articles [14-26]. The PRISMA diagram (Figure 1) shows the number of studies generated from the databases, included, and excluded, along with the reasons for the exclusion.

Study Characteristics

All studies included in this review were designed retrospectively, except one prospective study [18], with a duration ranging from 2002 to 2019. A total of 532 patients with a minimum mean age of 59.4 ± 9.78 years and a maximum mean age of 79 ± 6.9 years were analyzed. The studies were performed in several countries, including China [16], Brazil [17], India [14], USA [25,26], Pakistan [20], Austria [15], Spain [18], Sweden [22], Scotland [24], Canada [23], and Germany [19,21]. The included patients were overweight with a mean BMI above 27 kg/m^2^. The classification of bone loss was categorized in all studies based on the AORI classification, whereas types II and III at the femoral and tibial sides were seen. Besides, the ASA was used only in six studies [14,15,19,21,24,26]. Most of the studies had a comparable group, such as revision TKA versus primary TKA groups, femoral head allograft versus tantalum metal cones, and cemented versus uncemented metaphyseal sleeves groups, as shown in Table 1.

**Table 1:** Baseline population characteristics. * Prospectively. AORI: Anderson Orthopaedic Research Institute; ASA: American Society of Anesthesiologists; BMI: body mass index; FU: follow-up; TKA: total knee arthroplasty; TMC: tantalum metal cones; rTKA: revision total knee arthroplasty; NA: not reported in the original study.

First author, year, country	Study aim	Study duration	Number of patients	Gender		Mean age (Years)	Mean BMI (kg/m^2^)	ASA grade, N (%)	Anderson classification “AORI”, N (%)
				Female, N (%)	Male, N (%)				
Hippalgaonkar et al. (2024), India [14]	Investigate the clinical outcomes, complications, and efficacy of metaphyseal sleeves	December 2016 to January 2019	29	21 (72.4%)	8 (27.6%)	62.6 (SD: 7.8)	29.9 (SD: 5.51)	Grade 1: 4 (13.8%); Grade 2: 22 (75.9%); Grade 3: 3 (10.3%)	Type 2 and Type 3, "Type 2 not specified"
Eder-Halbedl et al. (2023), Austria [15]	Examine the survival of first-generation tantalum metal cones (TMC)	January 2011 to December 2015	100 (14 (14%) had died unrelated to the surgical intervention, and 6 (6%) had lost follow-up)	44 (55%)	36 (45%)	65.3	29.7	Mean: 2.17	Type 2a: 19 (24%); Type 2b: 28 (35%); Type 3: 33 (41%)
Liu et al. (2023), China [16]	Investigate the outcomes of the metaphyseal sleeve reconstruction of bone defects	2014 to 2019	43 patients (44 knees). Primary TKA: 7; rTKA: 37	33 (76.7%)	11 (23.3%)	65.9	27.2	NA	Type 2a: 18 (31.6%); Type 3b: 30 (52.6%); Type 3: 9 (15.8%)
Minamoto et al. (2022), Brazil [17]	Evaluate the midterm outcomes of knee reconstructions with trabecular TMC	July 2008 to November 2014	11. Primary TKA: 1; rTKA: 6; 2^nd^ revision: 4	8 (72.7%)	3 (27.3%)	67.54 (SD: 10.74)	29.78 (SD: 6.36)	NA	Tibial: Type 2a: 1 (9.1%); Type 2b: 6 (54.5%); Type 3: 3 (27.3%). Femoral: Type 3: 2 (18.2%)
Floria-Arnal et al. (2021), Spain [18]	Determine whether cement should be used to achieve fixation of the tibial tray	January 2010 to December 2013*	60. Group 1 (cemented sleeves): 30; Group 2 (uncemented sleeves): 30	32 (53.3%)	28 (46.7%)	Median: 75	29.8	NA	Type 2b
Lycke et al. (2021), Germany [19]	Examine the clinical outcome of rTKA with metaphyseal bone defects and instability using metaphyseal sleeves	May 2011 to March 2019	16. Group 1 (rTKA): 4; Group 2 (primary TKA): 12	9 (56.3%)	7 (43.8%)	G1: 76.5 (SD: 12). G2: 79 (SD: 6.9)	NA	Grade 2: 1 (6.3%); Grade 3: 15 (93.8%)	Type 3
Gill et al. (2020), Pakistan [20]	Assess the reliability of metaphyseal sleeves in dealing with massive bone defects to provide stability	2011 to 2019	36 patients (43 knees). Group 1 (rTKA: 31; Group 2 (primary TKA): 12	15 (41.6%)	21 (58.3%)	59.4 (SD: 9.78)	NA	NA	Type 2b and Type 3
Wirries et al. (2019), Germany [21]	Analyze the midterm results and implant survival of osteointegrative sleeves	2011 to 2014	47	39 (83%)	8 (17%)	67.2 (SD: 9.2)	30.6 (SD: 5.4)	Grade 2: 21 (45.6%); Grade 3: 24 (52.2%); Grade 4: 1 (2.2%)	Femoral: Type 1: 10 (21.3%); Type 2a: 13 (27.7%); Type 2b: 22 (46.8%); Type 3: 2 (4.2%). Tibial: Type 1: 2 (4.3%); Type 2a: 20 (42.6%); Type 2b: 21 (44.7%); Type 3: 4 (8.5%)
Thorsell et al. (2018), Sweden [22]	Evaluate patients operated on with a TKA using metal metaphyseal sleeves	2003 to 2010	31. Group 1 (rTKA): 22; Group 2 (primary TKA): 9	20 (64.5%)	11 (35.5%)	69	28	NA	Femoral: Type 1: 12 (38.7%); Type 2: 3 (9.6%); Type 3: 16 (51.6%). Tibial: Type 1: 9 (29%); Type 2: 5 (16%); Type 3: 17 (53.1%); "Type 2 not specified"
Sandiford et al. (2017), Canada [23]	Compare the clinical outcomes and complications between femoral head allografts and TMC	2002 to 2008	44. Group 1 (femoral head allograft): 30; Group 2 (TMC): 14	19 (43.2%)	25 (56.8%)	G1: 66; G2: 71	NA	NA	Group 1: Femoral: Type 2a: 9 (20.5%); Type 2b: 2 (4.5%); Tibial: Type 2a: 13 (29.5%); Type 2b: 6 (13.6%). Group 2: Femoral: Type 2a: 1 (2.2%); Type 2b: 7 (16%). Tibial: Type 2a: 4 (9%); Type 2b: 2 (4.5%)
Bugler et al. (2015), Scotland [24]	Assess the short-term outcomes of the use of metaphyseal sleeves in rTKA	2008 to 2013	34 patients (35 knees)	14 (43%)	20 (57%)	72	30.2	Grade 1: 3 (9%); Grade 2: 20 (57%); Grade 3: 12 (34%)	Femoral: Type 1: 17 (49%); Type 2: 16 (46%). Tibia: Type 1: 20 (57%); Type 2: 13 (37%); Type 3: 2 (6%); "Type 2 not specified"
De Martino et al. (2015), USA [25]	Determine the mid-term reoperation rates, radiologic findings of osseointegration	2005 to 2008	18 patients (18 knees)	12 (66.6%)	6 (33.3%)	73	29	NA	Femoral: Type 2b: 3 (16.6%); Type 3: 10 (55.5%). Tibia: Type 2b: 3 (16.6%); Type 3: 9 (50%)
Kamath et al. (2015), USA [26]	Assess the mid-term clinical and radiographic results of porous tibial cone implantation	2002 to 2015	63 patients (66 knees)	36 (57%)	27 (43%)	67	33	Mean: 2.4	Type 2a: 17 (25.7%); Type 2b: 25 (37.8%); Type 3: 24 (36.6%)

Intervention Details of the Studies

Table 2 demonstrates that the included studies had a mean follow-up ranging from 3.25 to nine years. A total of eight studies implanted metaphyseal metal sleeves, either stemmed or stemless, as a fixation technique, while the tantalum metal cones (TMCs) were implanted in five studies. The most commonly adopted functional assessment tools were the Knee Society Score (KSS), the Western Ontario and McMaster Universities Osteoarthritis Index (WOMAC), and range of motion (ROM). On the other hand, the visual analog scale (VAS), the Short Form Survey (SF-12 and SF-36), the Hospital for Special Surgery (HSS) knee score, and the Oxford Knee Score (OKS) have been utilized in a few studies. Moreover, the health-related quality of life questionnaire (EuroQol (EQ-5D)) was used in one study [22], and another one used the University of California, Los Angeles (UCLA) activity scale [23]. Also, the patient satisfaction was assessed only in three studies [16,23,24]. Furthermore, the radiographs evaluated the osseointegration, periprosthetic radiolucencies, and implant loosening postoperatively in all studies.

**Table 2:** Details of the metaphyseal fixation techniques in rTKA. KSS: Knee Society Score; OKS: Oxford Knee Score; ROM: range of motion; SF-12: Short Form-12; WOMAC: The Western Ontario and McMaster Universities Osteoarthritis Index; KOOS: Knee Injury and Osteoarthritis Outcome Score; EuroQol (EQ-5D): Health-related quality of life questionnaire; VAS: visual analog scale; HSS: Hospital for Special Surgery knee score; PJI: prosthetic joint infection; UCLA: University of California, Los Angeles; NA: not reported in the original study.

First author, year, country	Mean follow-up (years)	Revision knee surgery location, N (%)	Fixation technique (intervention)	Functional assessment tool	Indications for primary TKA, N (%)	Reason for revision, N (%)
Hippalgaonkar et al. (2024), India [14]	Minimum: 4	Left knee: 17 (58.6%), right knee: 12 (41.4%)	Porous-coated metaphyseal sleeves	KSS and OKS, radiographic assessment	26 (89.7%) osteoarthritis, 3 (10.3%) rheumatoid arthritis	18 (62.1%): aseptic loosening; 7 (24.1%): PJI; 3 (10.3%): instability; 1 (3.4%): periprosthetic fracture
Eder-Halbedl et al. (2023), Austria [15]	6.1	Left knee: 44 (55%), right knee: 36 (45%)	First-generation tantalum metal cones (TMC)	KSS, VAS, WOMAC index, ROM, and radiograph	75 (94%) tibia and 76 (95%) femur bone defects	16 (20%) PJI; 35 (45%) aseptic loosening; 14 (17%) instability; 11 (14%) osteolysis; 2 (3%) periprosthetic fractures; 1 (1%) osteomyelitis
Liu et al. (2023), China [16]	4.6 (SD: 1.47)	NA	Metaphyseal metal sleeves	KSS, HSS, WOMAC index, SF‐12, VAS score, and radiographic assessment	Osteoarthritis and rheumatoid arthritis cause tibia and femur bone defects	13 (35.1%) infection, 7 (18.9%) aseptic loosening; 7 (18.9%) instability; 5 (13.5%) postoperative pain; 3 (8.1%) stiffness; 1 (2.7%) periprosthetic fracture; 1 (2.7%) mechanical failure
Minamoto et al. (2022), Brazil [17]	7.28 (SD: 1.88)	Left knee: 7 (63.6%), right knee: 4 (36.4%)	Metaphyseal tantalum metal cones 10 (90.9%), Trabecular metal cones 1 (9%)	NA	NA	1 (9%) pain; 1 (9%) instability; 2 (18%) aseptic loosening; 2 (18%) infections
Floria-Arnal et al. (2021), Spain [18]	Maximum: 5	Left knee: 20 33.3%), right knee: 40 (66.7%)	Titanium alloy porous-coated metaphyseal sleeves and 75 mm-long uncemented stems	KSS knee and functional scales, WOMAC index, SF-12 health survey, and radiographic assessment	Defects in the femur and tibial bones	NA
Lycke et al. (2021), Germany [19]	G1: Median: 2.5; G2: Median: 6.63	NA	Metaphyseal sleeves	KSS, HSS, radiograph, and ROM	Severe metaphyseal bone defects and varus deformity	Major tibial and femoral bone deficiency situation and instability
Gill et al. (2020), Pakistan [20]	5.42 (SD: 2.24)	NA	Press-fit stemmed metaphyseal sleeves	KSS, ROM, and radiograph	Large metaphyseal bone defect, compromised collateral ligaments	NA
Wirries et al. (2019), Germany [21]	5	Left knee: 28 (59.6%), right knee: 19 (40.4%)	Cementless stemmed, partially poro-coated metaphyseal sleeves	KSS, WOMAC, and radiograph	NA	39 (62.9%) aseptic loosening, 4 (6.5%) instability, 2 (3.2%) malrotation of the implants, 17 (27.4%) septic loosening
Thorsell et al. (2018), Sweden [22]	7.4	NA	Metal metaphyseal sleeves with stems in 26 cases and without stems in 5 cases.	ROM, KOOS EuroQol (EQ-5D), and radiograph	16 (51.6%) primary osteoarthritis, 10 (32.2%) inflammatory arthritis, 4 (13%) post-traumatic osteoarthritis, 1 (3.2%) instability	8 (25.8%) infection, 7 (22.5%) aseptic loosening, 6 (19.4%) instability, 1 (3.2%) periprosthetic fracture
Sandiford et al. (2017), Canada [23]	G1: 9; G2: 7.23	NA	Femoral head allograft and trabecular metal cones	OKS, WOMAC, SF-12, the UCLA activity score, and radiograph	NA	37 (84%) aseptic loosening; 2 (4.5%) instability; 2 (4.5%) infection; 2 (4.5%) periprosthetic fracture
Bugler et al. (2015), Scotland [24]	3.25	Left knee: 16 (46%), right knee: 19 (54%)	Metaphyseal sleeves	KSS, OKS, SF-12, and radiograph	NA	16 (45%) aseptic loosening; 9 (26%) polyethylene wear; 6 (17%) malalignment; 2 (6%) instability; 2 (6%) unexplained pain
De Martino et al. (2015), USA [25]	6	Left knee: 6 (33.3%), right knee: 66.6%)	Tantalum metal cones	KSS, ROM, and radiograph	14 (77.7%) osteoarthritis, 4 (22.2%) rheumatoid arthritis	5 (27.7%) aseptic loosening, 13 (72.2%) second-stage reimplantation for deep infection
Kamath et al. (2015), USA [26]	5.8	Left knee: 32 (48%), right knee: 34 (52%)	Porous tantalum tibial metaphyseal cones	KSS, tibiofemoral alignment, and radiograph	10 (16%) rheumatoid arthritis, 7 (11%) inflammatory arthritis	26 (41.3%) deep infection, 15 (23.8%) aseptic loosening, 10 (15.9%) severe tibial osteolysis, 3 (4.8%) periprosthetic fracture, 12 (19%) instability

Regarding the primary and revision TKA indications, osteoarthritis and rheumatoid arthritis were the most reported reasons for primary TKA. Additionally, aseptic loosening, instability, pain, periprosthetic fracture, and PJI were the predominant complications that led to rTKA surgery. However, two studies did not report the reason for the revision [18,20].

Outcomes of Metaphyseal Fixation Techniques in rTKA

Among the 13 studies, the survival rate was reported in eight studies. The metaphyseal cone implant showed a survivorship rate of 91% in five years [23]. The metaphyseal sleeve implant demonstrated a higher survivorship rate of 100% in five years [18]. Outcome measures varied across studies (KSS, WOMAC, OKS), preventing direct quantitative comparison of functional results. Therefore, we provided a descriptive summary of patient-reported outcome scores. By contrast, mid-term implant survival across studies ranged from 86% to 100% over follow-up intervals of four to eight years.

Moreover, a stable alignment, osteointegration, and successful union have been observed in the radiographic results of the included studies; however, two studies showed mild radiolucencies after metaphyseal fixation using cones and sleeves, as presented in Table 3 [21,26].

**Table 3:** Outcomes of metaphyseal fixation techniques in rTKA. KSS: Knee Society Score; OKS: Oxford Knee Score; ROM: range of motion; SF-12: Short Form-12; WOMAC: The Western Ontario and McMaster Universities Osteoarthritis Index; KOOS: Knee Injury and Osteoarthritis Outcome Score; EuroQol (EQ-5D): Health-related quality of life questionnaire; VAS: visual analog scale; HSS: Hospital for Special Surgery knee score; TMCs: tantalum metal cones; rTKA: revision total knee arthroplasty; UCLA: University of California, Los Angeles; FU: follow-up; Pre: pre-operative; Post: post-operative; NA: not reported in the original study.

First author, year, country	% Survival	Functional outcome						Conclusion
		KSS (mean score)	VAS (mean score)	WOMAC (mean score)	ROM (mean score)	Radiograph	Others (mean score)	
Hippalgaonkar et al. (2024), India [14]	86.2% in 4 years	Pre: 57.97 (SD: 6.9); Post: 73.59 (SD: 4.8) (P<0.0001)	NA	NA	Pre: 55.4° Post: 102.2°	Stable positioning and alignment of the components	OKS Pre: 15.86 (SD: 2.8); Post: 30.66 (SD: 2.8) (P<0.0001)	Metaphyseal sleeves offer a viable solution for managing severe bone loss in rTKA, providing stable fixation, restoring joint line kinematics, and facilitating stress distribution to the metaphyseal region
Eder-Halbedl et al. (2023), Austria [15]	95% in 8 years	Clinical: Pre: median: 52 (IQR 22); Post: median: 90 (IQR 20.0). Functional: Pre: median: 45 (IQR 26); Post: median: 77 (IQR: 30.0) (P=0.001)	Pre: median: 7 (IQR 1); Post: median: 2 (IQR 3)	NA	Pre: median: 90° (IQR 14.0°) Post: median: 110° (IQR 34.0°) P=0.001	Stable 75 (94%) tibia and 76 (95%) femur TMCs. Risks were noted in 4 (5%) on the tibial side and 2 (2.5%) on the femoral side	NA	TMC demonstrates a secure fixation for treating severe femoral and tibial metaphyseal bone defects during rTKA
Liu et al. (2023), China [16]	100% in 6.4 years	Clinical: Pre: 37.1 (SD: 19.7); Post: 86.5 (SD: 13.6). Functional: Pre: 32.7 (SD: 24.0); Post: 78.3 (SD: 15.6) (P<0.001)	Pre: 5.9 (SD: 2.0); Post: 1.2 (SD: 1.1)	Stiffness: Pre: 9.1 (SD: 4.2); Post: 1.6 (SD: 2.5). Function: Pre: 26.1 (SD: 15.6); Post: 19.50 (SD: 17.9). Pain Pre: 8.4 (SD: 3.6); Post: 5.3 (SD: 3.8) (P<0.001)	Pre: 72.61° (SD: 33.42°) Post: 108.52° (SD: 24.15°)	Tight integration between the metaphyseal metal sleeve and bone	HSS: Pre: 48.20 (SD: 13.74); Post: 83.59 (SD: 7.88)	Metaphyseal sleeves combined with cementless stems are an excellent and viable option for reconstructing knee bone defects
Minamoto et al. (2022), Brazil [17]	NA	NA	NA	NA	NA	Showed signs of osteointegration of the implant	NA	The tantalum metaphyseal cones provided efficient structural support to prosthetic implants
Floria-Arnal et al. (2021), Spain [18]	100% in 5 years	Clinical: G1: 77.5 (SD: 15.5); G2: 82.1 (SD: 8.3). Functional: G1: 78.1 (SD: 8.6); G2: 81.8 (SD: 7.2)	NA	Pain: G1: 4.3 (SD: 2.8); G2: 3.2 (SD: 2.2). Function: G1: 17.6 (SD: 12); G2: 12 (SD: 8.9). Stiffness: G1: 1.9 (SD: 1.3); G2: 1.6 (SD: 1.4)	NA	Satisfactory alignment except in one patient in group 1, and optimal bone integration into the implant	SF-12 physical G2: 45 (5.6); G1: 43.4 (7.7); SF-12 mental G2: 57 (7.5); G1: 52.7 (9.7)	Cementation of the tibial tray would not be required to achieve satisfactory mid-term results
Lycke et al. (2021), Germany [19]	NA	Clinical: G1: median: 53; G2: median: 72. Functional: G1: median: 55; G2: median: 82.5	NA	NA	G1: median: 95° G2: median: 112°	No radiological signs of aseptic loosening in all patients	HSS: G1: median: 73; G2: median: 84. Pain level: G1: Pre: median: 8; Post: median: 2. G2: Pre: median: 8.5; Post: median: 2.5	Metaphyseal sleeves in patients with bone defects are a suitable instrument with no negative impact on primary and revision arthroplasty outcomes
Gill et al. (2020), Pakistan [20]	NA	Pre: 36.21 (SD: 7.43); Post: 92.00 (SD: 5.66) (P<0.005)	NA	NA	Pre: 76.83 (SD: 14.07°) Post: 122.91 (SD: 4.84°) P<0.005	Union was achieved in all cases	NA	Metaphyseal sleeves showed excellent short to mid-term survivorship
Wirries et al. (2019), Germany [21]	93.6%	Clinical: Pre: 56.6 (SD: 16.4); Post: 72.8 (SD: 13.0). Functional: Pre: 47.8 (SD: 23.2); Post: 57.4 (SD: 21.4) (P<0.001)	NA	Pre: 155.8 (SD: 58.6); Post: 91.0 (SD: 52.5). Pain, stiffness, and activity all P<0.001	NA	23 (48.9%) no radiolucency; 11 (23.4%) radiolucency >1 mm. Among 11, 5 (10.6%) metaphyseal radiolucencies. 1 (2.12%) cumulated amount of lucent lines of 6.4 mm. 10 (21.3%) radiolucency <4 mm	NA	Cementless osteointegrative sleeves for metaphyseal fixation yielded continuous implant fixation even in cases with preceding revisions
Thorsell et al. (2018), Sweden [22]	97%	NA	Pain at rest: 13 (SD: 25). On movement: 30 (SD: 31)	NA	Flexion: 110 Extension: –3	Good osseointegration with no signs of progressive radiolucency or migration	KOOS: Pain: 61 (27) Other symptoms: 65 (23). Function in daily living: 47 (26). Function in sports and recreation: 10 (13). Knee-related quality of life: 44 (35). EQ-5D: 0.5 (0.4)	Titanium sleeves are a promising option in managing difficult cases with metaphyseal bone defects in TKA, providing a stable construct with a good medium-term radiographic outcome
Sandiford et al. (2017), Canada [23]	Group 1: 93% (95% CI, 77–98) for 5 years, 93% (95% CI, 77–99) for 10 years. Group 2: 91% (95% CI, 56–98) for 5 years	NA	NA	G1: 82.3 (9.5); G2: 84.6 (9.5)	NA	Union of the allograft was noted from 3 months and was complete in all cases at 6 months. Osseointegration of trabecular metal cones was reported at 3 months FU	OKS: G1: 80 (10.1); G2: 84 (10.1). UCLA score: G1: 5.8 (1.2); G2: 5.5 (1.2). Patient satisfaction: G1: 93 (14.9); G2: 95.2 (14.9)	No difference in pain, function, or repeat revision when comparing femoral head allografts and trabecular metal cones for severe bone defects during revision
Bugler et al. (2015), Scotland [24]	NA	Clinical: 81.3 (18.1); Functional: 58.1 (33.1)	NA	NA	100°	No evidence of osteolysis around or loosening of either the femoral or tibial prostheses	OKS: 34.0 (9.6); SF-12 Physical: 38.3 (10.8); SF-12 Mental: 47.1 (10.4); Satisfaction: 7.5 (2.3)	Good outcomes, objectively, functionally, and radiographically, using metaphyseal sleeves in revision knee arthroplasty
De Martino et al. (2015), USA [25]	NA	Clinical: Pre: 31; Post: 77 (P<0.001). Functional: Pre: 22; Post: 65 (P<0.001)	NA	NA	Flexion contracture: Pre: 6°; Post: 3°. Average flexion: Pre: 88°; Post: 105°	No evidence of loosening or migration of any implant	NA	Tantalum cones in rTKA provided secure fixation with excellent results at an average of 6 years FU
Kamath et al. (2015), USA [26]	93.9% at last FU	Pre: 55; Post: 80 (P<0.0001)	NA	NA	Flexion contracture: Pre: 4.2; Post: 2. Average flexion: Pre: 87.5; Post: 104.2	1 had progressive radiolucencies about the tibial stem and cone. 1 had complete radiolucencies about the tibial cone, concerning fibrous ingrowth	Tibiofemoral alignment: Pre: 2.1 valgus; Post: 4.9 valgus (P=0.008)	Porous tantalum tibial cones offer a promising management option for severe tibial bone loss with durable clinical results and radiographic fixation

Metaphyseal sleeves significantly improve the functional status of the knee [14,16,20,21]. Similarly, a notable enhancement in knee functional status was observed after the use of metaphyseal cones [15,25,26].

Complications and Challenges of Metaphyseal Fixation Techniques in rTKA

Table 4 summarizes intraoperative and postoperative complications for sleeves (n = 296) and cones (n = 206). In the sleeve group, 11 patients (3.7%) sustained intraoperative fractures, and one (0.3%) had an intraoperative wound infection. Postoperatively, sleeves were associated with three cases of aseptic loosening (1.0%), 15 infections (5.1%), nine fractures (3.0%), and two wound complications (0.7%); overall, seven patients (2.4%) required a re-operation.

**Table 4:** Complications of the metaphyseal fixation techniques in rTKA. G: group; PJI: periprosthetic joint infection; DVT: deep vein thrombosis; rTKA: revision total knee arthroplasty; NA: not reported in the original study.

First author, year, country	Intraoperative complications, N (%)	Postoperative complications					Need for re-operation or re-revision, N (%)
		Aseptic loosening, N (%)	Infections, N (%)	Fracture/crack, N (%)	Wound, N (%)	Others, N (%)	
Hippalgaonkar et al. (2024), India [14]	NA	0	4 (13.8%) PJI	0	2 (6.9%)	2 (6.9%): tibial stem tip pain	None
Eder-Halbedl et al. (2023), Austria [15]	0	2 (2.5%)	2 (2.5%) deep infections; 1 (1.25%) recurrent infection	2 (2.5%)	2 (2.5%) superficial wound	1 (1.25%) recurrent instability; 1 (1.25%) sec. patella resurface	8 (10%) re-operation; 6 (7.5%) re-revisions
Liu et al. (2023), China [16]	4 (9.3%) longitudinal fractures	0	1 (2.3%) superficial wound infection		0	1 (2.3%) distal tibia pain; 2 (4.65%) delayed healing	None
Minamoto et al. (2022), Brazil [17]	NA	0	0	0	0	1 (9%) subcutaneous hematoma; 1 (9%) erysipelas	4 (36.26%) re-revision; 2 (18.2%) re-operation
Floria-Arnal et al. (2021), Spain [18]	0	NA	G2: 1 (3.3%) superficial surgical wound infection	G1: 2 (6.6%) femoral; G2: 1 (3.3%) tibial	0	G1: 1 (3.3%) DVT and 1 (3.3%) seroma; G2: 1 (3.3%) DVT	None in both groups
Lycke et al. (2021), Germany [19]	NA	NA	NA	4 (25%)	NA	2 (12.5%) hematoma; 2 (12.5%) recurrent effusions; 1 (6.25%) patellar tendon rupture; 1 (6.25%) peroneal lesion; 4 (25%) delayed healing	NA
Gill et al. (2020), Pakistan [20]	3 (6.9%) Iatrogenic fracture of the tibial condyle	0	1 (2.3%) PJI	NA	NA	2 (4.6%) radiolucent lines	NA
Wirries et al. (2019), Germany [21]	3 (6.4%) lateral tibial bone loss	3 (6.4%)	2 (4.3%) deep PJI	NA	NA	NA	NA
Thorsell et al. (2018), Sweden [22]	NA	NA	4 (13%)	1 (3.2%) periprosthetic fracture	NA	1 (3.2%) skin necrosis; 1 (3.2%) wound rupture	7 (22.6%) re-operation
Sandiford et al. (2017), Canada [23]	NA	NA	NA	1 (2.3%) periprosthetic fracture in G1	NA	NA	1 (2.3%) re-revision in G1
Bugler et al. (2015), Scotland [24]	1 (3%) proximal tibial fracture; 1 (3%) early postoperative myocardial infarction; 1 (3%) superficial wound infection; 1 (3%) wound dehiscence	NA	NA	1 (3%) femoral condylar fractures	NA	1 (3%) varus instability; 3 (8.8%) patellofemoral symptoms	0
De Martino et al. (2015), USA [25]	NA	NA	2 recurrent infections	NA	NA	NA	2 re-operation
Kamath et al. (2015), USA [26]	2 (3.2%) infections	2 (3.2%)	7 (11%)	4 (6.3%)	NA	1 (1.6%) granuloma and superficial hematoma	15 (24%) re-operation

Additionally, out of 206 patients who underwent metaphyseal cone fixation, no intraoperative fractures were reported, and two intraoperative infections (1.0%) were noted. After surgery, cones had four instances of aseptic loosening (1.9%), 12 infections (5.8%), five fractures (2.4%), and two wound issues (1.0%). Eleven patients (5.3%) underwent re-revision, and 27 (13.1%) required re-operation.

Sleeves carried a higher intraoperative fracture rate (3.7% vs. 0%), whereas cones led to more repeat surgeries (13.1% vs. 2.4%).

Quality Assessment

**Figure 2:**
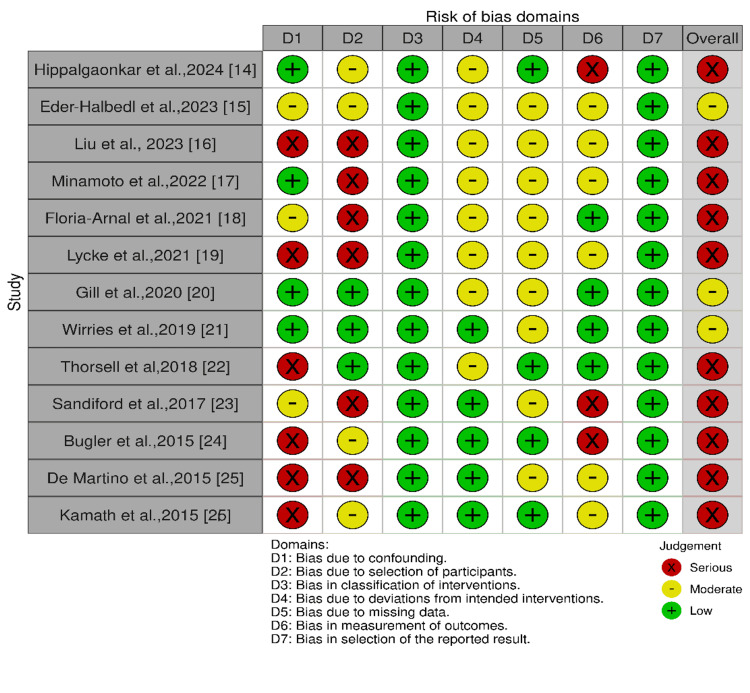
Risk of bias assessment using the ROBINS-I tool presented as a proportion of relevant studies (n = 13). ROBINS-I: Risk of Bias in Non-randomized Studies of Interventions.

The risk of bias assessment was assessed using the Cochrane Collaboration ROBINS-I tool, which revealed that 10 studies had a serious risk of bias [14,16-19,22-26], while three studies had a moderate risk of bias [15,20,21]. The most common sources of bias were confounding, participant selection, and intervention classification. The results of ROBINS-I quality assessments are reported in Figure 2, which was created with Robvis (Risk-of-Bias VISualization).

As illustrated in Figure 2, 10 of the 13 included studies were judged to have a serious overall risk of bias, primarily due to inadequate control of confounding variables and suboptimal participant selection, three studies showed a moderate risk, and none were rated as low risk. These biases tend to overestimate implant survival by privileging healthier cases.

Overall, implant survival ranged from 86% to 100% over follow-up intervals of four to eight years, but 10 of the included studies showed a serious risk of bias, primarily from unmeasured confounding, which may have spuriously inflated these survival figures.

Discussion

In light of the divergent complication profiles and implant survivorship rates observed across our cohort, we now explore the biomechanical and methodological factors that may drive these differences.

The number of rTKA surgeries is increasing due to the bone loss complication associated with surgery, affecting the positioning and alignment of the implanted parts [27]. Consequently, several fixation techniques were developed to manage bone loss during rTKA, including metaphyseal sleeves and metaphyseal cones. Additionally, the metaphyseal fixation techniques constitute the optimal therapy for AORI type IIb and type III bone defects [28]. Nonetheless, the literature reported a potential survival rate for metaphyseal sleeves and metaphyseal cones in the short term, with insufficient data in the long term. Therefore, this review was to present a comprehensive comparison of the clinical and radiological outcomes and complications of metaphyseal sleeves and metaphyseal cones in rTKA.

Despite a comprehensive search, most included studies were retrospective, single-center cohorts with variable follow-up, introducing moderate selection, detection, and reporting bias that should temper interpretation of fracture, infection, survivorship, and re-operation outcomes.

Fracture and Infection Rates

Unlike the metaphyseal cones, which are suitable for managing bone defects with fewer complications at mid-term follow-up [29,30], metaphyseal sleeves demonstrated a higher periprosthetic fracture and notable bone loss incidence [31,32]. In our cohort, metaphyseal sleeves carry a higher risk of intraoperative fracture (3.7% vs. 0% for metaphyseal cones), whereas postoperative infection rates remain comparable between metaphyseal sleeves (5.1%) and metaphyseal cones (5.8%).

Nonetheless, Bonanzinga et al., who conducted a systematic review aimed to evaluate and summarize the clinical outcomes of the use of metaphyseal sleeves in rTKA, observed that metaphyseal sleeves had low rates of aseptic loosening and intra-operative fractures (3.1%) with potential clinical outcomes at mid-term follow-up [33]. This is explained by the nature of metaphyseal sleeves, which have high-volume porosity (50-80%), leading to easier metaphysis bone growth [31].

Implant Survivorship

Moreover, our findings found that the metaphyseal sleeve implant demonstrated a higher survivorship rate compared to metaphyseal cones (100% for five years [18] versus 91% for five years [23]). This is consistent with another systematic review by Zanirato et al., who revealed a survivorship rate of 97.3% in the metaphyseal cones group and 97.8% in the metaphyseal sleeves group [34]. Also, Bonanzinga et al. reported a survivorship of 100% for metaphyseal sleeves [33].

Revisions and Re-operations

Furthermore, Jaibaji et al. found in a systematic review that assessed the mid-long-term clinical outcomes of metaphyseal fixation techniques that metaphyseal cones are associated with significantly higher rates of re-revision compared to the metaphyseal sleeves (P = 0.007), which mirrors our findings that cones required re-revision more than metaphyseal sleeves (5.33% vs. 0%), probably because of problems with cement, while metaphyseal sleeves caused more fractures during the initial surgery (3.7% vs. 0%). This was clarified by the use of cementation between metaphyseal cones and the implant, forming a possible failure of fixation, which was not found while using metaphyseal sleeves [35]. Although metaphyseal cones achieve excellent overall implant survival, their paradoxical increase in re-revisions underscores the need to optimize cementation strategies at the cone-implant interface.

Additionally, our review showed that metaphyseal cones had a higher rate of re-operations than those in metaphyseal sleeves (13.1 % versus 2.4%), which matches the results of another systematic review by Roach et al., as the re-operation rates were 18.7% for metaphyseal cones vs. 9.7% for metaphyseal sleeves [11].

Material Properties and Mechanisms

In terms of material properties, trabecular tantalum metaphyseal cones exhibits a random, thick‐strutted pore network (75-80% porosity; 2.5-3.9 GPa modulus; coefficient of friction ≈1), which deliver superior initial stability and fewer intraoperative fractures, whereas titanium metaphyseal sleeves (≈60% porosity; 330-390 µm struts; 3 GPa modulus) form a uniform lattice that concentrates stresses in slender elements and enhance osteointegration, but their thinner struts increase fracture risk during press-fit impaction [36,37].

Limitations

This review harbored several limitations. Several studies did not report key baseline characteristics, BMI was omitted in a few, and ASA grade was omitted in the majority, limiting our ability to ensure truly comparable patient cohorts. The exclusively retrospective observational design introduces selection bias. Based on our quality assessment, most studies were of low quality, and we identified no randomized controlled trials; positive‐result publication bias may further skew the literature toward overly optimistic survivorship and patient‐reported improvements. Finally, heterogeneity in the functional assessment tools used across studies restricts direct cross‐study comparisons and may undermine the reliability of our synthesized conclusions.

Recommendations and Future Directions

To close the most pressing evidence gap, we recommend multicenter randomized controlled trials that directly compare metaphyseal sleeves versus metaphyseal cones in patients with AORI type 3 bone defects, using standardized outcome measures such as KSS, validated patient-reported outcome instruments, and predefined radiographic loosening criteria, and following participants for a minimum of 10 years to capture both long-term prosthesis survivorship and late-onset complications. These trials should include prespecified, adequately powered sample-size calculations, control or stratify for key procedural variables, and embed targeted biomechanical substudies to elucidate device-specific failure mechanisms.

## Conclusions

Synthesizing these insights highlights key strategic considerations for metaphyseal fixation in rTKA and paves the way for targeted clinical and research recommendations. Our findings support a patient-tailored approach to metaphyseal fixation in rTKA. Metaphyseal sleeves deliver excellent mid-term survivorship (up to 100% at five years) and minimal re-revision, with a slightly elevated risk of intraoperative fracture. In contrast, metaphyseal cones provide superior initial press-fit stability and lower fracture incidence but exhibit higher re-operation and re-revision rates, underscoring the need to refine cementation techniques at the cone-implant interface. Clinically, surgeons should select fixation devices based on defect severity, bone quality, and patient comorbidities to balance osseointegration against mechanical safety.
